# Short-Term Associations Between a Government-Funded Sterilization Program for Owned Outdoor Dogs and Abandonment-Related Shelter Admissions in the Republic of Korea: A Nationwide Municipality-Level Panel Study

**DOI:** 10.3390/ani16203229

**Published:** 2026-10-14

**Authors:** HyungChul Rah

**Affiliations:** Department of Companion Animal and Plant Sciences, College of Future Convergence, Jeonju University, Jeonju 55069, Republic of Korea; hrah.joshua@jj.ac.kr

**Keywords:** dog abandonment, dog sterilization, owned outdoor dogs, dog population management, animal welfare, policy evaluation, panel data, difference-in-differences

## Abstract

Sterilization programs for owned outdoor dogs aim to reduce uncontrolled breeding and subsequent abandonment, but population-level evidence remains limited. This study examined short-term associations between the Korean government-funded sterilization program introduced in 2022 and shelter-recorded abandoned-dog admissions using annual data from 229 municipalities from 2019 to 2024 (1374 municipality-year observations). The outcome was shelter-recorded abandoned-dog admissions per 100,000 residents. Socioeconomic associations, program implementation, and implementation volume were evaluated using two-way fixed-effects (TWFE) and difference-in-differences (DID) models. Estimates were interpreted as ecological associations because parallel pre-intervention trends could not be fully established, program allocation was non-random, and eligible owned outdoor dog populations were unavailable. In the primary linear TWFE models, neither program implementation nor implementation volume was significantly associated with the shelter-recorded abandoned-dog admissions, and urban–rural-stratified analyses yielded similar null findings. Baseline-need-scaled sensitivity analyses produced positive associations in the pooled and urban samples, indicating sensitivity to denominator choice and model specification. Province-specific analyses were heterogeneous but inconsistent. Overall, the analyses provided no consistent evidence of a short-term reduction associated with the program. Future evaluations should measure coverage among eligible owned outdoor dogs, use longer follow-up, and evaluate sterilization within broader dog population management.

## Simple Summary

Owned outdoor dogs are a policy concern in the Republic of Korea because uncontrolled breeding may contribute to dog abandonment. Since 2022, the government has supported sterilization of owned outdoor dogs; however, whether the program has reduced abandonment at the population level remains unclear. Using annual municipality-level data from 229 municipalities from 2019 to 2024, this study examined whether shelter-recorded abandoned-dog admissions changed after program implementation and whether greater sterilization activity was associated with larger reductions. The analyses provided no consistent evidence of a nationwide short-term reduction. However, these findings do not demonstrate that sterilization is ineffective because the available data measured implementation volume rather than the proportion of eligible owned outdoor dogs sterilized. Future evaluations should measure the eligible dog population, sterilization coverage, reproductive status, owner participation, and local implementation practices.

## 1. Introduction

Dog abandonment is a major animal welfare, public health, and socioeconomic issue worldwide [1]. Many dogs enter shelters or remain in free-roaming populations because they have been abandoned, allowed to roam freely, or become lost [2]. Free-roaming or poorly supervised dogs may contribute to animal suffering, traffic accidents, livestock predation, wildlife impacts, zoonotic disease transmission, and conflicts with local communities. Consequently, reducing dog abandonment and improving responsible ownership have become important objectives of national animal welfare policies and international organizations, including the World Organisation for Animal Health (WOAH) and the International Companion Animal Management (ICAM) Coalition [3,4].

Among available dog population management strategies, surgical sterilization is one of the most widely implemented interventions [2]. Sterilization is expected to reduce unwanted reproduction, gradually decrease dog population growth, and ultimately reduce the number of dogs entering abandoned or free-roaming populations [5]. However, evidence regarding the population-level effectiveness of sterilization programs remains inconsistent. A systematic review concluded that fertility control was frequently associated with desirable outcomes, but the quality of evidence was generally limited because of heterogeneous study designs, inconsistent outcome measures, and limited use of appropriate comparison groups [2]. The need for rigorous evaluation of sterilization programs using robust epidemiological study designs has also been emphasized [6].

Conceptually, sterilization acts primarily through reproductive control. By reducing the number of reproductively active owned outdoor dogs, sufficiently broad and sustained sterilization coverage may reduce unintended litters and subsequent recruitment into abandoned or free-roaming dog populations. Animal-welfare and public-health benefits would therefore arise indirectly. Sterilization does not directly prevent owner relinquishment, loss, uncontrolled roaming, inadequate confinement, or irresponsible ownership; these pathways require complementary measures such as registration, permanent identification, owner education, roaming control, rehoming, and relinquishment prevention. Sterilization should therefore be regarded as one component of comprehensive dog population management rather than a complete strategy.

In the Republic of Korea, dog abandonment remains a major animal welfare concern despite ongoing government efforts to improve companion animal welfare [7,8]. Since 2022, the Korean government has implemented a nationwide publicly funded sterilization program targeting owned outdoor dogs, often referred to as yard dogs, with the stated objectives of reducing uncontrolled breeding and preventing future dog abandonment. In this study, “owned outdoor dogs” refers to dogs with identifiable owners that were primarily kept outdoors and fell within the eligibility criteria of the government-funded sterilization program. The term distinguishes these dogs from unowned, stray, or community dogs. Importantly, outdoor housing does not necessarily imply free roaming. Recent national data indicate that companion-animal ownership is widespread in the Republic of Korea. In the 2025 national companion-animal survey, 29.2% of households reported keeping companion animals, and dogs were kept by 80.5% of companion-animal-owning households [9]. However, nationally representative data do not provide a consistent distinction between dogs kept primarily indoors and owned dogs kept primarily outdoors, nor do they provide sterilization prevalence according to housing type. Consequently, national trends in the relative proportions or sterilization practices of indoor- and outdoor-kept dogs could not be quantified in the present study. Unlike many previous studies that focused on local sterilization projects or free-roaming dog populations, local governments nationwide have implemented this program under a common policy framework. Nevertheless, nationwide longitudinal data have not been used to examine its short-term, population-level association with dog abandonment.

Previous Korean studies have identified socioeconomic characteristics associated with dog abandonment using nationwide municipality-level data [8,10]. However, no study has examined whether the implementation volume of the owned outdoor dog sterilization program is associated with changes in dog abandonment after accounting for municipality-specific characteristics and year-specific shocks. In addition, the available administrative data do not include the number of eligible owned outdoor dogs, the number of reproductively intact dogs, or the proportion of the target population sterilized. Therefore, any analysis based on sterilized dogs standardized by the human population should be interpreted as an assessment of implementation volume rather than true program coverage.

Accordingly, this study examined short-term ecological associations between the government-funded owned outdoor dog sterilization program and shelter-recorded abandoned-dog admission rate across 229 municipalities from 2019 to 2024. Two-way fixed-effects (TWFE) regression and a difference-in-differences (DID) specification were used to account for time-invariant municipality characteristics and common year-specific shocks. Because program allocation was non-random and parallel pre-intervention trends could not be fully demonstrated, DID estimates were interpreted as adjusted ecological associations rather than definitive causal effects. Province-specific exploratory analyses assessed regional heterogeneity.

## 2. Materials and Methods

An ecological longitudinal panel study was conducted using annual municipality-level data from 229 local jurisdictions in the Republic of Korea from 2019 to 2024, yielding a balanced panel of 1374 municipality-year observations. The analysis-ready dataset is provided as Supplementary Data S1 and includes municipality identifiers, year, the shelter-recorded abandoned-dog admission rate, socioeconomic and demographic covariates, sterilization-program implementation volume, and the Treatment, Post, and DID indicators used in the statistical analyses.

The shelter-recorded abandoned-dog admission rate was selected as the study outcome. Statistics on dog abandonment in 229 local city, county, and district governments between 2019 and 2024 were obtained from the Korea Information Disclosure Portal (Open Government Information Disclosure System; https://www.open.go.kr/, accessed on 16 June 2026) and from animal abandonment reports published by the Korean Animal Welfare Association (KAWA) [11,12,13,14]. The study outcome was the annual number of dogs admitted to public animal shelters and administratively classified as abandoned, excluding dogs identified as lost and subsequently returned to their owners, as previously described [15]. The outcome was standardized per 100,000 residents and is hereafter referred to as the shelter-recorded abandoned-dog admissions per 100,000 residents or shelter-recorded abandoned-dog admission rate. This measure represents municipality-level shelter admissions rather than the true incidence of abandonment in the underlying dog population.

Independent variables comprised socioeconomic indicators, program implementation volume, and the DID interaction. Socioeconomic covariates were unemployment-benefit recipients per 100,000 residents, the proportion of residents aged 65 years or older, comprehensive income tax per capita, local income tax per capita, and population density. Program implementation volume was the annual number of owned outdoor dogs sterilized under the government-funded sterilization program per 100,000 residents. Because municipality-level counts of eligible owned outdoor dogs were unavailable, this measure was not interpreted as sterilization coverage.

The program targeted owned outdoor dogs meeting administrative eligibility criteria: dogs with identifiable owners that were primarily kept outdoors. Outdoor housing was conceptually distinct from roaming status, but individual confinement or roaming behavior was unavailable. Eligible owners applied through local administrative offices, and local governments selected beneficiaries and coordinated procedures with designated veterinary providers. Indoor-kept companion dogs were not part of the stated target population; however, the dataset did not include individual application records or details of how each municipality verified housing-status eligibility.

Treatment was coded as 1 for municipalities that implemented the government-funded owned outdoor dog sterilization program during the study period and 0 otherwise. Post was coded as 1 for observations from 2022 to 2024 and 0 for observations from 2019 to 2021. The DID interaction term was defined as Treatment × Post. The annual program indicator provided in Supplementary Data S1 was municipality-year specific and could therefore vary within a municipality over time, whereas the Treatment variable used in the primary DID analysis was an ever-treated indicator that remained constant within each municipality across the study period. The annual program indicator and sterilization counts were derived from municipality-level administrative records obtained through the Korea Information Disclosure Portal and reflected reported program activity in each municipality-year. Thus, within-municipality variation in the annual indicator represented reported annual implementation rather than a data-presentation artifact.

Data on unemployment-benefit recipients were obtained from unemployment-benefit payment status data held by the Korea Employment Information Center (https://eis.work24.go.kr, accessed on 30 June 2026) and standardized per 100,000 residents. The proportion of residents aged 65 years or older was collected from the Korean Statistical Information Service (https://kosis.kr, accessed on 13 June 2026). Comprehensive income tax per capita and local income tax per capita were collected as previously described [8,10]. Population density was calculated by dividing regional population by regional area, collected from the Korean Statistical Information Service.

Descriptive statistics were presented as means, standard deviations, and ranges. Pairwise correlations and variance inflation factors were examined for multicollinearity; values below 10 were considered acceptable.

The primary analyses used annual data for all 229 municipalities observed annually from 2019 to 2024. TWFE regression models accounted for unobserved time-invariant municipality characteristics and nationwide year-specific shocks through municipality and year fixed effects.

Three nationwide TWFE models were estimated. Model 1 examined socioeconomic associations with the shelter-recorded abandoned-dog admission rate. Model 2 estimated Treatment × Post to assess whether treated municipalities experienced a different post-2022 change than untreated municipalities; the main effects of Treatment and Post were absorbed by municipality and year fixed effects. Model 3 replaced the DID interaction term with the annual sterilized dogs per 100,000 residents to assess implementation volume.

Nationwide models used heteroskedasticity-robust standard errors clustered by municipality to account for serial correlation from repeated annual observations. Regression coefficients, clustered standard errors, 95% confidence intervals, and two-sided *p*-values were reported.

The DID specification was not treated as a definitive causal estimator. Program allocation was non-random, municipalities with greater pre-existing dog-management needs may have been more likely to implement or intensify the program, and only three pre-intervention years were available. Because parallel trends could not be fully established, DID estimates were interpreted as exploratory adjusted associations within the TWFE framework.

As a sensitivity analysis, fixed-effects Poisson models used annual shelter-admission counts as the outcome and log annual municipality population as an offset. Models included municipality and year fixed effects and the same socioeconomic covariates, with municipality-clustered robust standard errors. Results are reported as Poisson coefficients and incidence rate ratios (IRRs) with 95% confidence intervals. Because non-random allocation and time-varying confounding remained unresolved, these models were not interpreted causally. All 229 municipalities, including municipalities located in the seven metropolitan-level cities and one self-governing city, were retained in the nationwide analyses. For the province-specific exploratory analyses only, the seven metropolitan-level cities and one self-governing city were excluded because implementation of the owned outdoor dog sterilization program was limited in these jurisdictions. Province-specific analyses were therefore conducted for nine provinces (Gyeonggi-do, Gangwon-do, Chungcheongbuk-do, Chungcheongnam-do, Gyeongsangbuk-do, Gyeongsangnam-do, Jeollabuk-do, Jeollanam-do, and Jeju-do) to explore regional heterogeneity. These analyses used the same model specifications as the nationwide analyses and included municipality and year fixed-effects. They were considered exploratory because of their small sample sizes, multiple subgroup comparisons, and the use of conventional rather than municipality-clustered standard errors. In provinces where all municipalities implemented the program during the study period, the DID estimator could not be identified because no untreated municipalities were available for comparison.

Nationwide analyses were performed in R (version 4.6.1; R Foundation for Statistical Computing, Vienna, Austria) using the fixest package to estimate the TWFE models with municipality-clustered robust standard errors. Province-specific exploratory analyses were conducted using IBM^®^ SPSS Statistics^®^ (version 30.0; IBM Corp., Armonk, NY, USA). All statistical tests were two-sided, and *p*-values < 0.05 were considered statistically significant.

To assess urban–rural differences, municipalities classified as *si* (city) or *gu* (district) were categorized as urban and *gun* (county) municipalities as rural. TWFE-DID and implementation-volume models were estimated separately within each stratum using the same covariates, fixed effects, and municipality-clustered standard errors as the nationwide analyses. Annual mean shelter-recorded abandoned-dog admission rates from 2019 to 2024 were plotted by treatment status and urban–rural classification (Supplementary Figure S1). Because the pre-policy period overlapped the early COVID-19 pandemic and the post-policy period overlapped normalization of social activities, the figure was used only for descriptive comparison; monthly data were unavailable. These analyses were treated as sensitivity analyses rather than causal estimates.

An additional sensitivity analysis scaled annual outcomes and sterilization activity to each municipality’s mean shelter-recorded abandoned-dog admissions during 2019–2021. ScaledOutcome was annual admissions divided by the municipality-specific baseline mean, and ScaledSterilization was annual sterilized dogs divided by the same denominator. TWFE models used ScaledOutcome with either Treatment × Post or ScaledSterilization as the focal exposure, retaining the same covariates, fixed effects, and clustered standard errors. Because the denominator was derived from the pre-policy outcome, these models were interpreted as sensitivity analyses rather than alternative causal estimators. As an additional sensitivity analysis, a year-specific Treatment × Year model was estimated using ScaledOutcome, with 2021 specified as the reference year and never-program municipalities (*n* = 70), defined as municipalities that did not implement the program in any year during the 2019–2024 study period, as the comparison group. Interaction terms were estimated for 2019, 2020, 2022, 2023, and 2024, while the 2021 interaction was omitted as the reference category. The model included municipality and year fixed effects, the same socioeconomic covariates used in the primary TWFE models, and municipality-clustered robust standard errors. A joint Wald test was additionally performed for the 2019 and 2020 pre-policy interaction coefficients. This analysis was used to assess differential pre-policy temporal patterns and year-specific post-policy associations and was interpreted as a sensitivity analysis rather than as a definitive causal event-study design because only three pre-policy years were available and municipality-specific program initiation dates were unavailable.

## 3. Results

### 3.1. Characteristics of the Nationwide Panel

The nationwide dataset contained 1374 observations from 229 municipalities observed annually from 2019 to 2024 (Table 1). No duplicate municipality-year records or missing values were identified for the outcome variable, DID, or implementation-volume variables. The sample included both program and non-program municipalities.

Shelter-recorded abandoned-dog admission rates, sterilization implementation volume, and socioeconomic indicators varied substantially across municipalities (Table 1), providing both between- and within-municipality variation for panel analyses. Multicollinearity was low, with all variance inflation factors below 10.

### 3.2. Socioeconomic and Demographic Associations

None of the measured socioeconomic variables was significantly associated with the shelter-recorded abandoned-dog admission rate after adjustment for municipality and year fixed effects (Table 2). The proportion of residents aged 65 years or older showed the largest association but did not reach statistical significance (*β* = 17.06, clustered SE = 10.13, *p*-value = 0.094). Although the overall adjusted *R*^2^ was 0.860, the adjusted within *R*^2^ was only 0.005, indicating that the time-varying socioeconomic covariates explained little of the temporal variation within municipalities. These variables are therefore interpreted primarily as adjustment covariates rather than strong determinants of temporal change.

### 3.3. Program Implementation and Implementation Volume

The primary TWFE-DID model showed no significant post-2022 difference in shelter-recorded abandoned-dog admissions between program and non-program municipalities (*β* = 2.516, clustered SE = 12.956, 95% CI: −22.900 to 27.932, *p*-value = 0.846, Table 3), indicating no detectable short-term differential change associated with program implementation. Implementation volume was also not significantly associated with shelter-recorded abandoned-dog admissions (*β* = −0.042, clustered SE = 0.132; 95% CI: −0.301 to 0.217; *p*-value = 0.753). In the fixed-effects Poisson sensitivity analysis, the pooled DID association was positive (*β* = 0.186, IRR = 1.204, 95% CI: 1.078–1.345, *p*-value < 0.001), whereas implementation volume remained non-significant (*p* = 0.841). The discrepancy from the linear TWFE estimate indicates sensitivity to outcome scaling and model specification and should not be interpreted as evidence that sterilization increased abandonment.

### 3.4. Province-Specific Exploratory Analyses

Province-specific results are summarized in Table 4 and detailed in Supplementary Table S1. Among the five provinces in which DID was estimable, only Chungcheongbuk-do showed a significant negative DID estimate (*β* = −193.071, *p*-value = 0.012). DID estimates were non-significant in the other estimable provinces and could not be estimated in Gyeongsangnam-do, Chungcheongnam-do, Jeollabuk-do, and Jeju-do because all municipalities were treated.

Implementation-volume estimates varied across provinces, but the pattern was inconsistent and did not provide consistent evidence of program-associated reductions in shelter-recorded abandoned-dog admission rate.

### 3.5. Urban–Rural and Baseline-Need Sensitivity Analyses

The sample included 148 urban municipalities (81 treated, 67 untreated) and 81 rural municipalities (78 treated, 3 untreated). Supplementary Figure S1 shows annual trends by treatment status and urban–rural classification. Temporal patterns differed across strata, but annual data cannot isolate COVID-19-related changes from other contemporaneous factors; the figure was therefore descriptive rather than a formal test of pandemic effects.

In urban municipalities (Supplementary Table S2), the DID estimate was −7.425 (clustered SE = 12.945, 95% CI: −32.797 to 17.947, *p*-value = 0.566), and the implementation-volume coefficient was −0.092 (clustered SE = 0.175, 95% CI: −0.435 to 0.252, *p*-value = 0.601). In rural municipalities, the DID estimate was 12.261 (clustered SE = 72.924, 95% CI: −130.668 to 155.190, *p*-value = 0.866), and the implementation-volume coefficient was −0.103 (clustered SE = 0.154, 95% CI: −0.405 to 0.200, *p*-value = 0.505). Neither program implementation nor implementation volume was significantly associated with lower admissions within either stratum.

Baseline-need-scaled results differed from the primary analyses (Supplementary Table S3). In the pooled sample, DID was positive (*β* = 0.222, clustered SE = 0.044, 95% CI: 0.135–0.309, *p*-value < 0.001), and ScaledSterilization was positively associated with ScaledOutcome (*β* = 0.321, clustered SE = 0.143, 95% CI: 0.041–0.601, *p*-value = 0.025). Similar positive associations were observed in urban municipalities (DID: β = 0.196, *p*-value < 0.001; ScaledSterilization: *β* = 0.495, *p*-value = 0.001), whereas neither association was significant in rural municipalities (DID: *β* = 0.203, *p*-value = 0.472; ScaledSterilization: *β* = 0.207, *p*-value = 0.215). These findings indicate sensitivity to denominator and scaling. Full pooled, urban, and rural Poisson sensitivity results are provided in Supplementary Table S4.

In the year-specific Treatment × Year sensitivity analysis using ScaledOutcome, with 2021 as the reference year, the pre-policy interaction coefficients were significantly negative in 2019 (*β* = −0.441, clustered SE = 0.058, 95% CI: −0.554 to −0.328; *p* < 0.001) and 2020 (*β* = −0.147, clustered SE = 0.044, 95% CI: −0.232 to −0.061; *p* < 0.001). The joint Wald test for the 2019 and 2020 coefficients was also statistically significant (*F* = 30.65; *p* < 0.001), indicating differential pre-policy temporal patterns between treated and never-program municipalities. In contrast, the post-policy interaction coefficients were not statistically significant in 2022 (*β* = 0.004, 95% CI: −0.077 to 0.085; *p* = 0.915), 2023 (*β* = 0.081, 95% CI: −0.019 to 0.182; *p* = 0.111), or 2024 (*β* = 0.079, 95% CI: −0.019 to 0.176; *p* = 0.114) (Supplementary Figure S2). These findings provide evidence of differential pre-policy patterns and further support cautious interpretation of the DID estimates.

## 4. Discussion

This nationwide panel study found no consistent evidence that participation in the government-funded owned outdoor dog sterilization program or greater implementation volume was associated with a short-term reduction in the shelter-recorded abandoned-dog admission rate. Province-specific estimates were heterogeneous but inconsistent and should be considered exploratory. The very low within-model explanatory power further indicates that annual changes in measured socioeconomic covariates explained little of the temporal variation within municipalities, suggesting an important role for relatively stable local characteristics and administrative context.

These findings should not be interpreted as a strong causal evaluation. Methodologically, DID analysis requires credible parallel pre-intervention trends and adequate handling of non-random treatment allocation. Only three pre-intervention years were available, and the additional year-specific sensitivity analysis indicated differential pre-policy patterns between treated and never-program municipalities. Municipalities with greater underlying dog-management needs may also have been more likely to implement the program. The estimates are therefore best interpreted as short-term ecological associations from administrative data rather than definitive causal evidence that the program did or did not reduce dog abandonment.

The absence of a significant nationwide association should not be interpreted as evidence that surgical sterilization is inherently ineffective. Instead, the impact of sterilization on dog population outcomes depends on program coverage, duration, implementation quality, the structure of the underlying dog population, and the extent to which sources of population recruitment are addressed simultaneously. A systematic review of dog population management studies found that fertility control was frequently evaluated and often associated with desirable outcomes, but evidence strength was limited by heterogeneous designs, inconsistent outcome measures, inadequate comparison populations, and poor reporting quality [2].

The study measured implementation volume per 100,000 residents rather than coverage among eligible owned outdoor dogs. A municipality with many sterilization procedures per resident may still have low target-population coverage if its eligible dog population is large whereas fewer procedures may represent high coverage where the eligible population is small. Because municipality-level data on eligible owned outdoor dogs, reproductive status, sex and age were unavailable, biologically meaningful coverage could not be estimated.

The three-year post-policy observation period may also have been insufficient to detect population-level effects. Reductions in births may precede reductions in shelter admissions by several years because adult dogs remain in the population, sterilization coverage accumulates gradually, and abandonment may continue among existing owned-dogs. The findings should therefore be interpreted as evidence from early national implementation rather than as an assessment of long-term outcomes.

The fixed-effects Poisson sensitivity analysis produced a positive DID association in the pooled sample, whereas the corresponding linear TWFE estimate was not statistically significant. This discrepancy indicates sensitivity to outcome scaling and model specification. The positive Poisson estimate should not be interpreted as evidence that sterilization increased abandonment, because non-random program allocation, time-varying administrative activity, and other unmeasured differences between municipalities remain potential sources of confounding. Importantly, implementation volume itself remained non-significant in the Poisson model. It is important to distinguish surgical sterilization from comprehensive dog population management. Sterilization is specifically a reproductive-control intervention and directly addresses only one pathway through which dogs may enter abandoned or free-roaming populations: recruitment through reproduction. It does not by itself prevent relinquishment, loss, roaming, inadequate confinement, or irresponsible ownership. Consequently, the findings of this study apply specifically to the sterilization component of the Korean program and should not be interpreted as an evaluation of comprehensive dog population management as a whole. Systems modeling suggests that the greatest long-term reductions in free-roaming dog populations occur when fertility control is combined with responsible ownership interventions that reduce abandonment and roaming and increase adoption [16]. International guidance also supports comprehensive dog population management rather than reliance on a single intervention [3,4]. Accordingly, sterilization records should ideally be linked with permanent identification, dog registration, owner education, restrictions on uncontrolled roaming and breeding, and follow-up monitoring.

Province-specific estimates suggested possible regional heterogeneity, which is plausible given differences in dog population structure, ownership practices, rurality, access to veterinary services, administrative capacity, shelter operations, and community attitudes. However, these findings are hypothesis-generating because samples were small, conventional rather than municipality-clustered standard errors were used, multiple comparisons were performed, and DID was not estimable in provinces without untreated municipalities.

Nationwide administrative data enabled construction of a balanced six-year panel covering all 229 municipalities but were not designed for causal policy evaluation. Future studies should record municipality-specific implementation dates, the number of eligible owned outdoor dogs, sex and reproductive status, cumulative and lagged exposure, owner participation, and local implementation practices. These data would permit coverage-based evaluation and reduce uncertainty about program effectiveness.

An additional consideration is the distinction between service delivery and population-level impact. Reviews of dog population management interventions have shown substantial heterogeneity in the indicators used to evaluate programs and have emphasized that procedure counts alone do not capture changes in population size, welfare, ownership, recruitment, or other relevant outcomes [17]. Likewise, evaluations of subsidized small-animal veterinary services frequently report the number of animals reached, while denominator information and population-level outcome measures are often limited [18]. For the Korean program, future monitoring systems should therefore record the estimated number of eligible owned outdoor dogs in each municipality, the sex and reproductive status of sterilized dogs, repeat participation, and denominator-based annual and cumulative coverage.

Dog-population turnover also complicates interpretation of short-term changes in shelter intake. Demographic studies of free-roaming dogs indicate that births, deaths, immigration, emigration, acquisition, abandonment, and changes in ownership or roaming status jointly determine local population dynamics [19]. Sterilization directly addresses only recruitment through reproduction; therefore, continued inflows from abandonment, acquisition, movement, or uncontrolled breeding outside the program may offset or delay a measurable decline in abandoned-dog admissions even when fertility is reduced.

Several limitations should be considered when interpreting these findings. First, causal identification was limited by the available pre-policy data and by non-random program allocation. The additional year-specific Treatment × Year sensitivity analysis using ScaledOutcome identified significant differential pre-policy patterns in 2019 and 2020 relative to 2021. This finding further weakens a simple parallel-trends interpretation of the DID specification. However, because only three pre-policy years were available and ScaledOutcome itself uses a denominator derived from the 2019–2021 outcome history, this analysis should be viewed as evidence of model and denominator sensitivity rather than as a definitive formal test of the causal identification assumptions [20,21]. Municipalities with greater pre-existing dog-abandonment or population-management needs may also have been more likely to adopt or intensify the program, leaving the analyses vulnerable to time-varying confounding, reverse causation, and confounding by indication. In addition, if municipality-specific implementation timing varied after the national program was introduced, defining Treatment according to participation during the study period and applying a common Post period from 2022 to 2024 could mix municipalities with different lengths of actual exposure. Recent difference-in-differences literature emphasizes that variation in treatment timing and treatment-effect heterogeneity require careful interpretation and, where possible, estimators designed for multi-period or staggered adoption settings [20,22]. Accordingly, the DID estimate in this study should be regarded as an adjusted ecological association rather than a causal estimate of program effectiveness.

Second, exposure measurement did not permit estimation of true sterilization coverage. Program implementation volume was standardized by the human population rather than by the number of eligible owned outdoor dogs or reproductively intact dogs. Because the dog-to-human ratio and the prevalence of owned outdoor dogs are likely to differ substantially across urban, peri-urban, and rural municipalities, human-population standardization may not rank municipalities according to the proportion of the target dog population actually sterilized. The absence of municipality-level denominator data, as well as information on sex, reproductive status, age, and repeated participation, therefore prevents estimation of biologically meaningful annual or cumulative coverage [17,18].

Urban–rural and baseline-need sensitivity analyses further illustrated the importance of denominator choice and population structure. Urban–rural stratification did not materially change the linear TWFE findings: neither program implementation nor implementation volume was significantly associated with lower admissions. However, only three rural municipalities were untreated, substantially limiting the precision and comparability of the rural DID estimate.

Baseline-need scaling produced positive associations in the pooled and urban analyses, but these estimates should not be interpreted as evidence that sterilization increased abandonment. The denominator was derived from the pre-policy outcome and may be sensitive to baseline fluctuations and regression to the mean; it also does not address non-random allocation, local administrative activity, or changes in shelter-intake practices. The divergence across human-population-standardized, urban–rural-stratified, and baseline-need-scaled models shows sensitivity to denominator choice and model specification. Moreover, shelter admissions are not a direct measure of the eligible dog population, so baseline admissions are also an imperfect denominator.

The COVID-19 pandemic is another temporal consideration. The pre-policy period included the pandemic-affected years 2020–2021, whereas 2022–2024 overlapped with progressive normalization of social and administrative activities. Year fixed effects ac-count for common nationwide shocks but cannot remove municipality-specific pandemic effects related to urbanicity, administrative capacity, shelter operations, veterinary-service availability, or animal-control activity. Figure S1 therefore provides only a descriptive comparison of annual trajectories; monthly data were unavailable.

Third, the outcome represented dogs admitted to public shelters and administratively classified as abandoned after excluding lost dogs subsequently returned to their owners. It does not capture all abandoned, roaming, or poorly supervised dogs in the community and may be influenced by reporting behavior, capture effort, shelter accessibility, local intake capacity, and classification practices. Changes in shelter admissions may therefore reflect both changes in the underlying dog population and changes in local administrative or shelter operations.

Fourth, the three-year post-intervention period was relatively short for an intervention expected to act through reduced reproduction and gradual demographic turnover. Cumulative sterilization exposure and lagged associations could not be evaluated reliably with the available follow-up, and reductions in births may take several years to translate into lower shelter intake [19]. Longer follow-up is therefore needed before drawing conclusions about long-term population effects.

Fifth, the province-specific analyses were exploratory and subject to important inferential constraints: some provinces contained few municipalities, conventional rather than municipality-clustered standard errors were used, multiple subgroup comparisons were conducted, and DID was not estimable where no untreated municipalities were available. Because these analyses also excluded the seven metropolitan-level cities and one self-governing city owing to limited implementation, the regional findings should be viewed as hypothesis-generating and not generalized to those jurisdictions.

Time-varying local administrative capacity is another potential confounder. Municipalities that expanded sterilization may also have increased animal-control staffing, capture activity, enforcement, or shelter intake, potentially raising recorded admissions and sterilization volume simultaneously. Municipality fixed effects cannot fully account for these time-varying changes.

Despite these limitations, the study has several strengths, including a balanced six-year panel covering all 229 municipalities, municipality and year fixed effects, municipality-clustered robust standard errors in the nationwide models, transparent reporting of null and model-sensitive findings, and provision of the analysis-ready dataset. These features provide a national baseline for future evaluations with better target-population denominators, longer follow-up, municipality-specific implementation dates, and stronger quasi-experimental designs.

## 5. Conclusions

This nationwide panel study provided no consistent evidence that the Korean government-funded owned outdoor dog sterilization program was associated with a short-term reduction in shelter-recorded abandoned-dog admissions during its first three years. Estimates varied by urban–rural composition, denominator choice, and model specification, limiting causal interpretation. These findings do not demonstrate that sterilization is ineffective; rather, the available administrative data and human-population-standardized implementation measure were insufficient to establish coverage-based effectiveness. Future evaluations should measure sterilization coverage among eligible owned outdoor dogs, incorporate longer follow-up and municipality-specific implementation timing, address non-random program allocation, and evaluate sterilization within a broader dog population management strategy that includes registration, responsible ownership, education, movement control, rehoming, and long-term monitoring.

## Figures and Tables

**Table 1:** Characteristics of the nationwide municipality-level panel data.

**(A) Panel Characteristics**					
**Characteristic**	**Value**				
Municipalities	229				
Years	2019–2024				
Total observations	1374				
Balanced panel	Yes				
Municipality fixed effects	Yes				
Year fixed effects	Yes				
Cluster-robust SE	Municipality level				
**(B) Descriptive Statistics**					
**Variable**	**Observed Region**	**Mean**	**SD**	**Min**	**Max**
Shelter-recorded abandoned-dog admission rate	1374	290.154	298.866	2.691	2035.016
Unemployment-benefit recipients per 100,000 residents	1374	3090.352	772.116	737.448	5454.862
Population aged ≥65 years (%)	1374	23.635	8.939	7.600	47.500
Comprehensive income tax (KRW 1000 per capita)	1374	637.676	1052.923	98.035	12,521.363
Local income tax per capita (KRW 1000 per capita)	1374	342.437	676.903	46.083	10,491.807
Population density(persons/km^2^)	1374	3756.843	5894.010	18.805	26,316.198
Sterilized dogs per 100,000 residents	1374	47.193	134.831	0.000	2249.939

**Table 2:** Nationwide two-way fixed-effects (TWFE) estimates of socioeconomic and demographic factors.

Model	Variable	Estimate (*β*)	Clustered Standard Errors	*p*-Value	95% Confidence Intervals	*N*	Adjusted *R*^2^	Adjusted Within *R*^2^
Model 1: Nationwide TWFE	Unemployment-benefit recipients per 100,000 residents	0.006	0.019	0.746	−0.032 to 0.044	1374	0.86	0.005
Model 1: Nationwide TWFE	Population aged ≥65 years (%)	17.055	10.133	0.094	−2.822 to 36.933	1374	0.86	0.005
Model 1: Nationwide TWFE	Comprehensive income tax per capita	0.000	0.000	0.153	<−0.001 to <0.001	1374	0.86	0.005
Model 1: Nationwide TWFE	Local income tax per capita	0.000	0.000	0.605	<−0.001 to <0.001	1374	0.86	0.005
Model 1: Nationwide TWFE	Population density (persons/km^2^)	0.011	0.015	0.475	−0.018 to 0.040	1374	0.86	0.005

**Table 3:** Nationwide two-way fixed-effects estimates of program implementation and implementation volume.

Model ^†^	Variable	Estimate (*β*)	Clustered Standard Errors	*p*-Value	95% Confidence Intervals	*N*	Adjusted *R*^2^	Adjusted Within *R*^2^
Model 2: TWFE-DID	Treatment × Post (DID)	2.516	12.956	0.846	−22.900 to 27.932	1374	0.860	0.004
Model 3: Implementation volume	Sterilized dogs per 100,000	−0.042	0.132	0.753	−0.301 to 0.217	1374	0.860	0.005

^†^ Each row is from a separate model. All models adjust for unemployment-benefit recipients, population aged ≥65 years, comprehensive income tax, local income tax, population density, municipality fixed effects, and year fixed effects. Standard errors are clustered at the municipality level.

**Table 4:** Exploratory province-specific estimates of sterilization-program effects on shelter-recorded abandoned-dog admission rate.

Province	Municipalities	Observations	DID Estimate (SE)	*p*-Value	Adjusted *R*^2^	Program-Intensity Estimate (SE)	*p*-Value	Adjusted *R*^2^
Chungcheongbuk-do	11	66	−193.071 (73.204)	0.012 *	0.851	0.190 (0.300)	0.53	0.829
Chungcheongnam-do	15	90	NE	NE	NE	−0.036 (0.210)	0.864	0.784
Gangwon-do	18	108	81.892 (85.785)	0.343	0.865	0.171 (0.152)	0.263	0.866
Gyeonggi-do	31	186	2.864 (21.730)	0.895	0.975	−0.294 (0.146)	0.045 *	0.976
Gyeongsangbuk-do	22	132	79.195 (116.461)	0.498	0.75	−0.050 (0.128)	0.696	0.749
Gyeongsangnam-do	18	108	NE	NE	NE	−0.187 (0.077)	0.017 *	0.846
Jeju-do	2	12	NE	NE	NE	−1.497 (1.229)	0.29	0.939
Jeollabuk-do	14	84	NE	NE	NE	0.240 (0.371)	0.52	0.594
Jeollanam-do	22	132	34.639 (229.514)	0.880	0.657	−0.337 (0.295)	0.256	0.661

* *p* < 0.05, NE, not estimable because all municipalities within the province were treated and no untreated comparison group was available.

## Data Availability

The data presented in this study are available within Supplementary Materials.

## Supplementary Materials

The following supporting information can be downloaded at: https://www.mdpi.com/article/10.3390/ani16203229/s1, Data S1: Analysis-ready nationwide municipality-level panel dataset; Table S1: Province-specific two-way fixed-effects estimates of socioeconomic/demographic factors, program implementation, and implementation volume; Table S2: Urban–rural-stratified sensitivity analyses of the association between the owned outdoor dog sterilization program and shelter-recorded abandoned-dog admissions; Table S3: Baseline-need-scaled sensitivity analyses using municipality-specific mean 2019–2021 shelter-recorded abandoned-dog admissions as the denominator; Table S4: Fixed-effects Poisson sensitivity analyses of the association between the owned outdoor dog sterilization program and shelter-recorded abandoned-dog admissions; Figure S1: Annual mean shelter-recorded abandoned-dog admissions, 2019–2024, by treatment status and urban/rural municipality classification; Figure S2: Year-specific Treatment × Year coefficients for ScaledOutcome, with 2021 as the reference year.
